# Development of Gum-Acacia-Stabilized Silver Nanoparticles Gel of Rutin against *Candida albicans*

**DOI:** 10.3390/gels8080472

**Published:** 2022-07-27

**Authors:** Mohammed H. Alqarni, Ahmed I. Foudah, Aftab Alam, Mohammad A. Salkini, Magdy M. Muharram, Nikolaos E. Labrou, Piyush Kumar

**Affiliations:** 1 Department of Pharmacognosy, College of Pharmacy, Prince Sattam Bin Abdulaziz University, Alkharj 11942, Saudi Arabia; ahmedfoudah11@gmail.com (A.I.F.); a.alam@psau.edu.sa (A.A.); m.salkini@psau.edu.sa (M.A.S.); 2 Department of Microbiology, College of Science, Al-Azhar University, Nasr City, Cairo 11884, Egypt; magdymuharram@azhar.edu.eg; 3 Laboratory of Enzyme Technology, Department of Biotechnology, School of Food, Biotechnology and Development, Agricultural University of Athens, 75 Iera Odos Street, GR-11855 Athens, Greece; lambrou@aua.gr; 4 Department of Chemistry, Indian Institute of Technology, NH-44, PO Nagrota, Jagti, Jammu 181221, India; piyushniper@gmail.com

**Keywords:** rutin, *Candida albicans*, silver nanoparticles, biocompatibility, gum acacia, anti-fungal

## Abstract

*Candida spp*. is one of the most causative pathogens responsible for fungal infections. It is often a hospital-acquired form of sepsis with a very high number of deaths. Currently, the most effective anti-fungal agents are based on polyenes or echinocandins. However, long-term treatments or repeated use of these anti-fungals lead to therapy limitations. Current research is urgently needed to overcome existing challenges for antimicrobials from natural sources. This study aims to determine the anti-fungal activity of rutin, which has the advantage of increasing the therapeutic value. Because of its low solubility in water and oils, rutin is limited in use. To address these constraints, we encapsulated rutin in a nanocarrier system. Silver nanoparticles (SNPs) and gum acacia (GAs) are emerging as attractive components and are widely studied as biologically safe nanomaterials/carrier systems for various drugs. Still, they are barely investigated as nano-sized vectors for the targeted delivery of rutin. In the present work, GA stabilised SNPs of rutin were successfully formulated and evaluated. It was later incorporated into carbapol 940 gels and formed SNP gels. Rutin-SNPs were developed with a consistent size in the nano range of 59.67 ± 44.24 nm in size, 0.295 ± 0.014 polydispersity index (PDI), and −11.2 ± 6.66 mV zeta potential. The drug released was found to be 81. 26 ± 4.06% in 600 min by following zero-order kinetics. The rutin-SNP gel showed considerable activity against *C. albicans* skin candidiasis at MIC 1.56 g/mL. The developed formulation was biocompatible. This first-ever interdisciplinary study suggests that the rutin-SNPs gel could play a vital role in drug resistance in this fungal pathogen.

## 1. Introduction

The increased fungi resistance to conventional antimicrobials poses a significant public health threat worldwide [1]. *Candida spp.* is an opportunistic fungus that can be pathogenic and cause a clinical manifestation such as candidiasis under predisposition conditions. Among the different species, *Candida albicans* (*C. albicans*) is one of the most prevalent pathogens that causes many fungal diseases. It constantly colonizes the oral cavities of immunocompromised individuals and causes psychological and physical harm [2]. An essential factor in the pathogenesis of candidiasis is the formation of biofilms, as *f* can form them on both inert and biological surfaces. In particular, living prostheses and catheters have the best surface condition for forming *Candida* biofilms [3]. *C. albicans* pathogens isolated in bloodstream infections associated with catheters and has a high mortality and morbidity rate [4].

Anti-fungals, mainly oral, transdermal/topical, and intravenous, treat various fungal infections. However, when the antimicrobials are taken from the oral route, it is reported to be more toxic to the human body. In addition, commonly used anti-fungals contain a wide range of components, including azoles, echinocandin, and polyenes [5]. Azole, such as clotrimazole, and amino compounds, such as nafticin and terbinafine, promise to treat skin and oral skin disease with synthetic drugs [6]. However, their frequent use, inadequate drug concentration at the site of disease, increased exposure of healthy areas to treatment, higher dosages required, associated side effects, increased frequency of administration, and poor patient compliance, further leads to underprivileged treatment outcomes [7] and the progress of resistance [8]. Candida species are naturally resistant to most of the commonly prescribed anti-fungal drugs. In addition, if the treatment continues, it leads to the development of multidrug-resistant (MDR) strains, which complicate the therapy [9,10,11]. In addition, these drugs cannot reach the target site, which may lead to incomplete elimination of the infection. To solve this problem, using natural origins as anti-fungals could be a practical approach [12].

Natural anti-fungal drugs may be a feasible solution to this problem. To gain further insight into these limitations, we selected rutin as an anti-fungal agent in the present study. Rutin is a subset of flavonoid glycosides present in some typical plants, which has nutritional importance [13]. Anti-inflammatory, antibacterial, anti-allergic, anti-cancer, and antioxidant activities are present in rutin [14,15]. Due to its enormous pharmacological potential which has shown great therapeutic potential in various diseases, including microbial diseases. Thus, the use of rutin is beneficial compared to other flavonoids because it is not toxic [16]

However, rutin has low solubility, stability, and bioavailability, which inhibits potential therapeutic effects in different diseases. These further limit their therapeutic potential for treating disease [17]. New drug delivery systems were developed for rutin to overcome these disadvantages and limitations. Furthermore, resistance mechanisms could be avoided by encapsulating antimicrobial bioactive components within nanoparticles [18,19,20].

Due to their high electrical conductivity, oxidative catalytic activities, and antibacterial activity, silver nanoparticles (SNP) are of great interest in the field of metal nanoparticles [21]. Since SNPs can be used in pharmaceuticals and for other purposes, scientists focus on producing them with biogenic agents and using them as antimicrobial, antioxidant, anticancer, antiviral, and pharmaceutical agents to treat many diseases [22]. Gum acacia (GA) with a high polysaccharide content is obtained as a sticky exudate from the stems and branches of acacia plants [23]. In this study, GA was used as a dispersal and reduction agent during the synthesis of silver nanoparticles. GA directly reduced silver nitrates to silver nanoparticles without any other additive [24].

**Figure 1:**
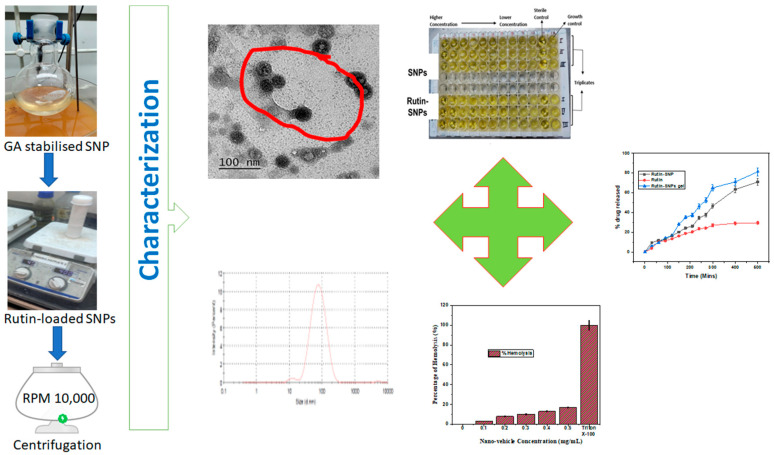
Schematic representation of workflow.

As natural polymers in GA can adjust the synthesis nanoparticles with biomedical potential, it was planned in this study to synthesize AgNPs using GA. Subsequently, to explore the antimicrobial potential of GA stabilised rutin-loaded Silver nanoparticles (rutin-SNPs) were further investigated for their physicochemical properties to evaluate the in vitro anti-fungal efficacy. The workflow of the present work is shown in Figure 1.

## 2. Results and Discussion

### 2.1. FTIR Analysis

**Figure 2:**
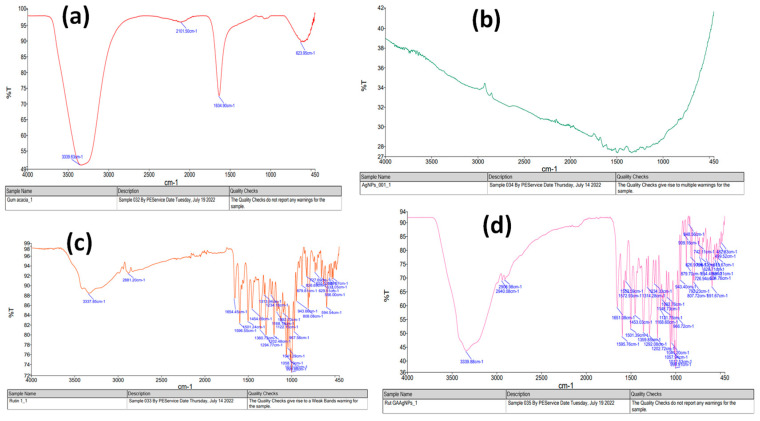
FTIR of (**a**) Gum-acacia; (**b**) Silver nanoparticles; (**c**) Rutin; (**d**) Rutin-SNPs.

The FTIR spectrum shown in Figure 2a–d shows the composition of the spectrum. As shown in Figure 2a, Gum-acacia solution presented strong vibrational peaks at 1600 cm^−1^ (C=O stretching) and the band that presented at 3337 cm^−1^ indicates the presence of a hydrogen-bonded -OH group. The peaks which appeared at 3337, 2800–3000, and 1654 cm^−1^ in the FTIR spectrum of rutin belong to-OH, alkane groups (–CH, –CH_2_, –CH_3_), and C=O stretching vibrations, respectively, as shown in Figure 2c,d. These peaks also appeared in the FTIR spectrum of AgNPs (Figure 2b).

### 2.2. Morphology

**Figure 3:**
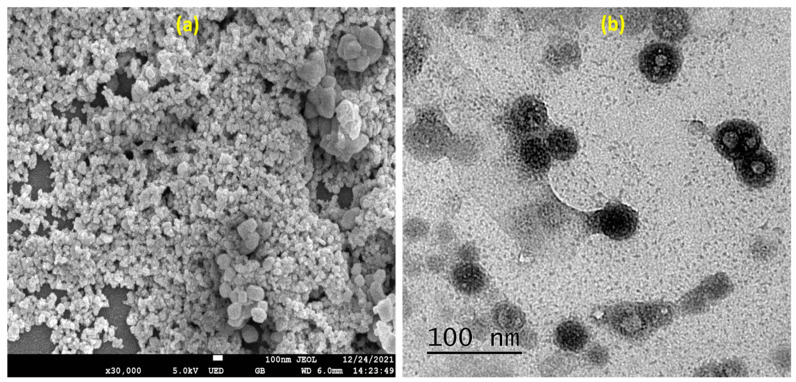
Morphology of Rutin-SNPs (**a**) FE-SEM; (**b**) HRTEM.

The topography study essentially confirmed the surface texture of prepared composites and emphasized morphology prediction. The anti-fungal potential of nanoparticles is also influenced by their shape [25]. The surface morphology of the rutin-SNPs was determined using SEM (Figure 3a). As shown, the obtained nanoparticles are almost spherical and have a smooth surface for rutin-SNPs. The morphology study supports the previously published work [26]. The morphological analysis of the rutin-SNPs was further evaluated by TEM. The photos show that the rutin SNPs are distinct and homogeneous, with approximately spherical shapes (Figure 3b). The image is similar to previous research work published [27]. The size is also comparable to the one observed in the dynamic light diffusion studies.

### 2.3. Size, Polydispersity Index (PDI), and Zeta Potential (ZP)

**Figure 4:**
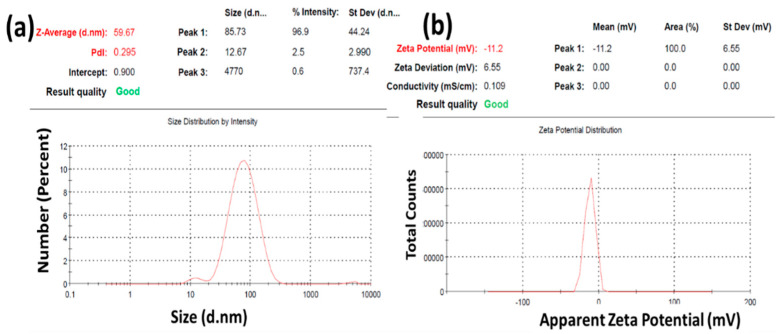
Characterization of rutin-SNPs for (**a**) Size, PDI; (**b**) zeta size.

Figure 4a depicts the nanoparticles’ dynamic light scattering (DLS) spectra. DLS results showed that newly developed rutin-SNPs are nano in range. The mean average hydrodynamic size of the doped materials is 59.67 ± 44.24 nm. The polydispersity index value was observed to be less than 0.295 ± 0.014. As expected, the average particle size obtained by TEM was much larger than that obtained from DLS. It may be due to the processing of the sample for TEM without any further dilution or modification. The results from TEM were therefore found to be unreliable due to artifacts in sample preparation. In DLS, the diameter becomes smaller when the sample is size fractionated because the autocorrelation functions are measured on monodisperse dispersions [28]. Previous work reflects a similar range of DLS and PDI [29]. The ZP was observed to be −11.2 ± 6.66 mV. The negative ZP indicates colloidal stability in the formed water suspension, as shown in Figure 4b. The studies mentioned above have shown that rutin SNPs can be successfully prepared with different charging ratios.

### 2.4. % Entrapment Efficiency (EE)

The potential carrier of drugs must have a high load capacity and structural integrity. Therefore, studying the maximum drug load and relative conditions is crucial. In the present study, rutin encapsulation was reported to be 56.04 ± 2.34%. It was concluded that SNP was a suitable drug carrier for rutin.

### 2.5. Physicochemical Evaluation of Rutin-Loaded SNPs into Carbopol Gel Formulations

It was observed that the gels formulated by carbopol and rutin-SNPs were clear and uniformly consistent. This might be due to the nature of the gelling agent carbapol being synthetic, which might be of the purest quality giving a clear appearance. Table 1 shows the rheological characteristics of the gels. Formulations were evaluated for pH and were found to be in the acceptable range of 6.0 ± 0.3, similar to the skin’s natural pH. Since the pH of the mixture was within the typical range of the pH of the skin, it did not irritate during application. The spread of the gel formulation was found to be 9.914 ± 0.39 mm. It has been shown that the gels are easy to spread with a small amount of cutting and have good spreadability. The viscosity of the rutin-SNPs was 2456 cps.

### 2.6. In Vitro Drug Release

**Figure 5:**
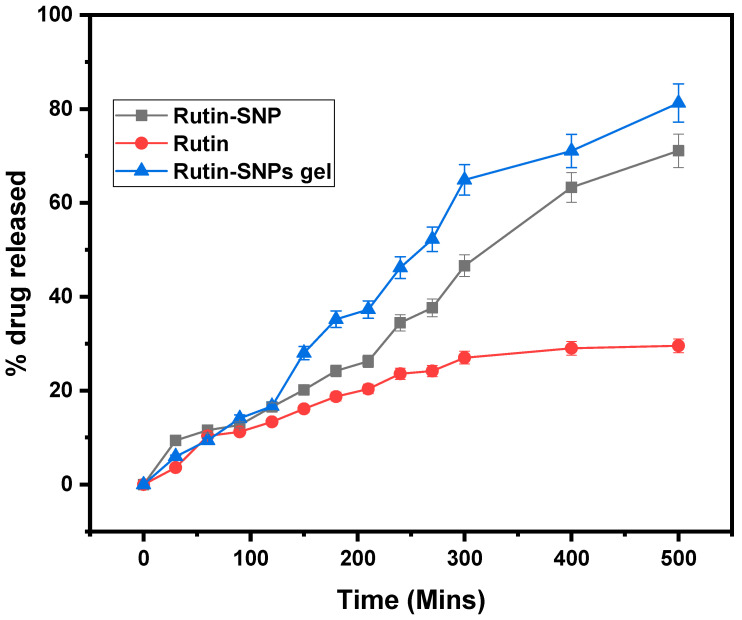
Comparative in vitro drug release study of rutin, rutin-loaded SNPs, and rutin-loaded SNPs gel for 600 min.

The release of rutin from SNP loaded with rutin, SNP gel loaded with rutin, and rutin powder was evaluated at pH 6.4 for 600 min. As shown in Figure 5, only 16.101 ± 0.802% of the rutin was released from the bare rutin powder in 150 min. In the case of SNP-rutin NPs, the drug released was reported to be 20.147± 1.007% in 150 min. On the contrary, rutin was released within 150 min from SNP gel by more than 27.99 ± 1.3%. At the end of 600 min, the drug released was found to be 29.55 ± 1.4% (bare rutin), 71.088 ± 3.55 (rutin-loaded SNPs), and 81.26 ± 4.06 (rutin-loaded SNPs gel). Finally, the in vitro dissolution studies indicated that the formulation of rutin in SNPs in carbapol gel could enhance the dissolution properties. After this, a release kinetics study was carried out, and the r^2^ value is given in Table 2. The drug release pattern of rutin-loaded SNPs gel followed the Hixson Crowell model with r^2^ 0.9813, the highest among others. It means that there is a strong correlation between drug release from the particle and surface area, and the particle’s diameter area may be due to its small size.

### 2.7. Hemolysis

**Figure 6:**
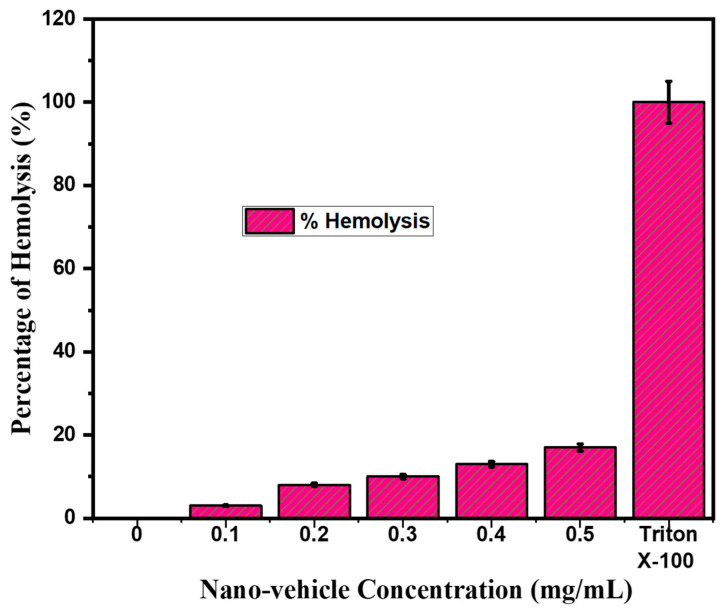
Hemolysis percentages of the rutin-loaded SNPs gel.

A hemolysis assay was performed to assess the toxicological effects of the SNPs gel nanoparticles on human erythrocytes (RBCs). It is owing to its widespread acceptance as a potential vehicle for the targeted delivery of therapeutics, as shown in Figure 6. The hemolysis caused by rutin-SNPs was found to be in the range of 3–15%. Our hemolysis results are consistent with those recently reported by Choi et al. (2011) [30]. It showed that our newly synthesized nanoparticles are safe and biocompatible. Hence, it can be concluded that our newly synthesized rutin-SNPs gel is highly hemocompatible in nature.

### 2.8. Anti-Fungal Activity

**Figure 7:**
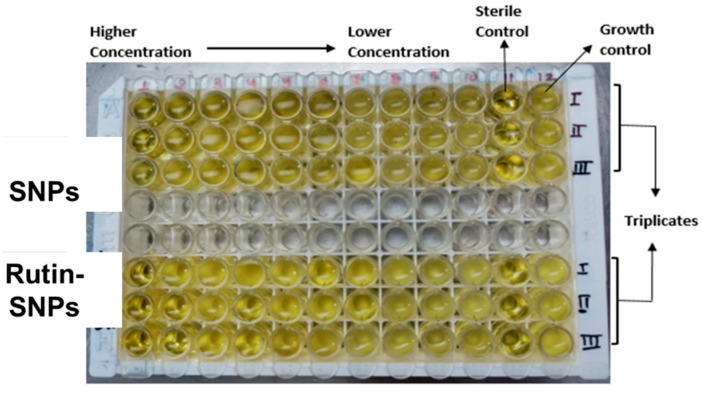
Microtiter plate wells showing MIC of SNPs and Rutin-SNPs.

Based on the results, rutin-loaded SNPs gel showed higher MIC values than nanoparticles for the selected fungi species. This suggested that the silver-GA solution was less effective as an anti-fungal agent than rutin-loaded SNPs. The MIC for SNPs gel was reported to be 6.25 μg/mL, whereas rutin-loaded SNPs gel showed 1.56 μg/mL against *Candida albicans,* as shown in Figure 7. These differences can be caused by the capsizing effects of rutin on formed SNPs. Capping causes significant changes in AgNP antimicrobial activity [31]. In conclusion, rutin-AgNP showed excellent antifungal activity at MIC low and did not induce drug resistance for *C.albicans.* The results suggested that rutin-SNPs had excellent anti-fungal activity against *C. albicans* and offered a potential anti-fungal agent for wound healing.

## 3. Conclusions

In this work, a new strategy for the preparation of herbal components-based SNP gels has been developed. The properties of the resulting materials were characterized to assess the effect of SNPs and GA on rutin. After characterization, it was further incorporated into the carbapol 940 base gels to form an SNP rutin gel. Rutin SNP gel shows significant activity against *C. albicans* skin candidiasis. The formulation of gels has shown its effectiveness, such as increasing the penetration of drugs into the skin, reduce dose, minimize frequent application and avoid adverse reactions to creams, solutions, or liposomes. The SNP gel encapsulation of rutin was undertaken to better address the low specificity and efficiency problems encountered in general ointments. The results showed that this formulation could be promising in the topical delivery of clove oil for the treatment of fungal infections.

## 4. Materials and Methods

Rutin and silver nitrate were purchased from Sigma Aldrich, USA. Acacia gum and Carbopol 940 were purchased from Loba Chemie (P) Ltd., Mumbai. Di-sodium hydrogen phosphate dihydrate (Sigma-Aldrich) and sodium dihydrogen orthophosphate dihydrate (Merck) were used to prepare 0.1 M phosphate buffer (PB) for buffer solution preparation. Nutrient agar media, Sabouraud dextrose agar media, nutrient broth, and Sabouraud dextrose broth were purchased from Himedia, India. Analytical grade solvents and chemicals were used in their purest form and were not further purified.

### 4.1. Preparation of Silver Nanoparticles Using Gum Acacia

In 100 mL of deionized water, 0.5 g of acacia gum (GA) was dissolved. A solution of 0.5 g AgNO_3_ in 100 mL DI water was prepared. In a tube, 5 mL of AgNO_3_ containing GA was added. This mixture was kept in an autoclave for 2 min at a temperature of 120 °C and a pressure of 15 psi. The clear off-white solution showed that silver nanoparticles had formed [32].

#### Formation of Rutin-Loaded SNPs

Rutin is not water-soluble. To make it soluble, we added DI water and ethanol at a ratio of 3:7. For 15–20 min, the solution was stirred. After 20 min of stirring, 15 mL of GA-SNPs were added to this solution, which was then continuously stirred for 24 h at room temperature. There was no evidence of drug precipitation. After that, the solution was centrifuged for 45 min at 10,000 rpm. The rutin, GA-stabilized SNPs were analyzed. UV-Vis absorption spectroscopy was used to evaluate the drug concentration in the supernatant.

### 4.2. Characterization

#### 4.2.1. Infrared Spectral Analysis

Infrared spectroscopy is one of the most important analytical methods, providing a sensitive instrument for detecting certain functional groups of polymers and drugs [33,34]. FTIR spectroscopy (PerkinElmer FT-IR) was used to determine drug carrier interactions in physical mixtures. During four scanning, spectral ranges of 500–4000 cm^−1^ were recorded in the IR absorption mode with a resolution of 2 cm^−1^.

#### 4.2.2. Morphology

The surface shapes of the rutin SNPs were examined with a scanning electron microscope (SEM; JEOL JSM-6490LV). To prepare the samples for measurement, they were air dried and then sputtered with gold. A transmission electron microscope (TEM, Jeol, JEM -1000) with an accelerated voltage of 100 Kv was used to examine the morphology of the rutin SNPs. Samples for TEM analysis were prepared by placing a small amount of the rutin-SNPs dispersion on a carbon-coated grid and allowing it to dry for about three minutes. This ensured that the sample would adhere to the grid, and filter paper was used to remove excess dispersion [35,36,37,38].

#### 4.2.3. Size, Polydispersity Index (PDI), and Zeta Potential (ZP)

Rutin SNPs were measured for particle size (PS), polydispersity index (PDI), and zeta potential (ZP) at 25 °C using a zeta sizer (Nano ZS, Malvern Instruments Corp, UK) after diluting the dispersion to a sufficient amount with deionized water. Each measurement was performed in triplicate [39].

#### 4.2.4. % Entrapment Efficiency (EE)

Rutin concentration was estimated using an ultrafiltration approach by measuring absorbance at 424 nm and using the Lambert–Beer law to determine the encapsulation efficiency (EE) of NPs using the following equation [40]:

%EE = (Total amount of rutin-amount of rutin in the supernatant) Total amount of rutin × 100(1)

#### 4.2.5. Preparation of Gel Formulation

An appropriate amount of Carbopol 940 was added to 100 mL distilled water. It was then allowed to hydrate and expand at room temperature for two hours to achieve a uniform mixture. After stirring at 800 rpm for 60 min, a few milliliters of triethanolamine were added dropwise to neutralize the formulation. The mixture was stirred until a clear gel was formed [41].

#### 4.2.6. Incorporation of Rutin-Loaded SNPs into Carbopol Gel

Optimized SNPs containing rutin were mixed into the 0.75% (*w*/*w*) carbopol gel with an electrical mixer (25 rpm, 2 min), and the carbopol gel was obtained.

#### 4.2.7. Physicochemical Evaluation of Rutin-Loaded SNPs into Carbopol Gel Formulations

The prepared gel was further evaluated for physical parameters such as color, appearance, consistency, pH, Viscosity, and Homogeneity [42].

#### 4.2.8. In Vitro Drug Release

In vitro release studies were performed to understand the release patterns by applying sink conditions [43,44]. Briefly, approximately 10 mm (MWCO 14,000 Da) dialysis bags were filled with 1 mL rutin SNP. The dialysis bag is knotted at both ends, immersed in a 40-mL bottle filled with pH 6.4 PBS phosphate buffer (PBS), and placed in a mechanical mixer at 37 °C and 100 rpm. At the specified time interval, the sample solution is removed and replaced with a fresh PBS solution (pH 6.4) maintained at the same temperature. The corrosion concentrations of the triplicate samples were quantified at 424 nm by UV spectroscopy using the corresponding blank samples. The data obtained with the bare and rutin SNPs were therefore kinetically analyzed using several mathematical models with the correlation coefficient (R^2^).

#### 4.2.9. Hemolysis

A previously described method was used to determine the percentage of hemolysis [45]. Sheep blood was washed thrice with autoclaved phosphate buffer salt (PBS, pH 7.4) and centrifuged at 2800 rpm for 5 min. Rutin SNPs were diluted with PBS at a concentration of up to 0.05 to 0.5 mg/mL for each sample. Erythrocyte suspension (0.2 mL) was added to 1.8 mL of each sample and incubated at 37 °C for 30 min. Then the samples were centrifuged at 3000 rpm for 10 min. Spectrophotometric measurements of the supernatant of each sample at different concentrations were performed to determine hemoglobin release. To achieve 0% and 100% hemolysis, 0.2 mL of RBC suspension was added to 1.8 mL of PBS and distilled water. The degree of hemolysis was calculated using the following equation:

%Hemolysis = (At − Ac)/(Ax − Ac) × 100(2)

#### 4.2.10. Anti-Fungal Activity

Double serial dilution in a nutrient medium and Sabouraud dextrose broth made it possible to evaluate the MICs of SNPs, rutin SNPs, against the Candida albicans strains. MIC was performed on microlitre sterile plates. In brief, the Ag nanoparticles and rutin-SNP stocks solution was prepared in water to ensure complete solubility at a concentration of 1 mg/mL. A total of 100 µL of nutritional and sabotaged Dextrose broth were distributed in wells 1 to 10. SNPs and rutin-SNPs, (100 µL) were added to the first well. The solution was serially diluted from well 1 to well 10, while 100 µL was discarded from well 10. Then, 100 µL of fungal suspension was added to all dilution ranges from well 1 to well 10. The overnight fungal suspension (100 µL) was dispensed in well 11, and 100 µL of sterile broth was added to positive control or growth control. In comparison, 200 µL of sterile Nutrient Broth and Sabouraud Dextrose broth in well 12 served as negative or sterility control. The plate was incubated at 37 °C for 24 h. After incubation, the absorbance of each well was measured using an ELISA reader (Erba) at 640 nm wavelength. The procedure above was performed for each microbial strain. The sample concentration and standard that inhibited 50 percent of bacterial growth were determined for all microorganisms. All the tests were performed in triplicate to minimize the error [46].

## Figures and Tables

**Table 1:** Physicochemical characteristics of rutin-SNPs formulations.

Clarity	Homogeneity	pH	Viscosity (cps)	Spreadability	Extrudability
Clear	Homogenous	6.0 ± 0.3	2456	9.914 ± 0.39	19.19 ± 0.95

**Table 2:** Release kinetics data of rutin-loaded SNPs.

r^2^ Value	Zero-Order Model	First Order	Higuchi Model	Korsmeyer–Peppas Model	Hixson Crowell Model
Rutin-loaded SNPs gel	0.946	0.8876	0.863	0.814	0.9813

## Data Availability

Not applicable.
